# Swim Training Affects on Muscle Lactate Metabolism, Nicotinamide Adenine Dinucleotides Concentration, and the Activity of NADH Shuttle Enzymes in a Mouse Model of Amyotrophic Lateral Sclerosis

**DOI:** 10.3390/ijms231911504

**Published:** 2022-09-29

**Authors:** Karol Cieminski, Damian Jozef Flis, Katarzyna Patrycja Dzik, Jan Jacek Kaczor, Mariusz Roman Wieckowski, Jedrzej Antosiewicz, Wieslaw Ziolkowski

**Affiliations:** 1 Doctoral School, Poznan University of Physical Education, Królowej Jadwigi 27/39 Street, 61-871 Poznan, Poland; 2 Department of Pharmaceutical Pathophysiology, Faculty of Pharmacy, Medical University of Gdansk, Dębinki 7 Street, 80-211 Gdansk, Poland; 3 Department of Animal and Human Physiology, University of Gdansk, J. Bazynskiego 8 Street, 80-308 Gdansk, Poland; 4 Laboratory of Mitochondrial Biology and Metabolism, Nencki Institute of Experimental Biology, L. Pasteura 3 Street, 02-093 Warsaw, Poland; 5 Department of Bioenergetics and Physiology of Exercise, Faculty of Health Sciences, Medical University of Gdansk, Dębinki 1 Street, 80-211 Gdansk, Poland; 6 Department of Rehabilitation Medicine, Faculty of Health Sciences, Medical University of Gdansk, Aleja Zwycięstwa 30, 80-219 Gdansk, Poland

**Keywords:** ALS, neurodegeneration, muscle metabolism, exercise, nicotinamide adenine nucleotides, monocarboxylate transporters

## Abstract

In this study, we aim to verify whether swim training can improve lactate metabolism, NAD^+^ and NADH levels, as well as modify the activity of glycolytic and NADH shuttle enzymes and monocarboxylate transporters (MCTs) in skeletal muscle of amyotrophic lateral sclerosis (ALS) mice. ALS mice (SOD1G93A) (*n* = 7 per group) were analyzed before the onset of ALS, at first disease symptoms (trained and untrained), and the last stage of disease (trained and untrained), and then compared with a wild-type (WT) group of mice. The blood lactate and the skeletal muscle concentration of lactate, NAD^+^ and NADH, MCT1 and MCT4 protein levels, as well as lactate dehydrogenase (LDH) and malate dehydrogenase (MDH) activities in skeletal muscle were determined by fluorometric, Western blotting, liquid chromatography-MS3 spectrometry, and spectrometric methods. In the untrained terminal ALS group, there were decreased blood lactate levels (*p* < 0.001) and increased skeletal muscle lactate levels (*p* < 0.05) as compared with a WT group of mice. The amount of nicotinamide adenine dinucleotides in the ALS groups were also significantly reduced as well as LDH activity and the level of MCT1. Swim training increased lactate levels in the blood (*p* < 0.05 vs. ALS TERMINAL untrained). In addition, cytosolic MDH activity and the cMDH/LDH 2.1 ratio were significantly higher in trained vs. untrained mice (*p* < 0.05). The data indicate significant dysfunction of lactate metabolism in ALS mice, associated with a reduction in muscle anaerobic metabolism and NADH transporting enzymes, as well as swim-induced compensation of energy demands in the ALS mice.

## 1. Introduction

Amyotrophic lateral sclerosis (ALS) is an incurable, chronic neurodegenerative disease characterized by the selective death of motoneurons in the motor cortex, brainstem, and spinal cord, which control any muscle action [1]. Approximately 90% of ALS cases are sporadic (sALS), with unknown etiology, and the remaining are genetically determined (fALS). However, in clinical terms, both forms are identical. 

Significant reductions in aerobic and anaerobic energy metabolism, enhanced oxidative stress, increased proteolytic proteins, and atrophy of muscle fibers in skeletal muscle of ALS mice have been observed [2,3,4,5,6,7,8,9].

The disruption of skeletal muscle glycolytic metabolism in ALS mice develops as the disease progresses. The influence of swim training on lactate metabolism is still an unexplored phenomenon in the skeletal muscles of individuals with ALS.

Lactate (LA) is considered to be a waste product of the Embden–Meyerhof–Parnas pathway, known as glycolysis [10]. A reduction in pyruvate to LA, with the participation of reduced nicotinamide adenine dinucleotide (NADH), is catalyzed by lactate dehydrogenase (LDH) (pyruvate + NADH + H+ ↔ Lactate + NAD^+^) (see graphical abstract). However, NADH derived from glycolysis can alternatively react with oxaloacetate (OAA) in the cytosol (cytoplasmic malate dehydrogenase (cMDH): oxaloacetate + NADH + H^+^ → Malate + NAD^+^) and under the malate form, hydrogen can be transported into the mitochondria by NADH transport enzymes (the malate-aspartate shuttle system (MAS)) [11]. In this situation, pyruvate does not turn into LA but preferentially enters the mitochondria, where it is oxidized to CO_2_ and H_2_0 [12]. This mechanism of NADH oxidation can be identified by the ratio of cMDH to LDH activity. The higher the ratio of these two enzymes, the greater the role of MAS in the transport of NADH to the mitochondria and the recovery of the NAD^+^ pool in this way. As mentioned earlier, LA in skeletal muscle cells can be converted to pyruvate and further oxidized in the mitochondria (see graphical abstract). The conversion of LA to pyruvate is also catalyzed by lactate dehydrogenase (LDH) (LA + NAD^+^ → pyruvate + NADH + H^+^) [13]. 

It is worth noting that nicotinamide adenine dinucleotides are substrates or products of reactions catalyzed by dehydrogenases and also critical compounds in the course of ALS disease. Enhancing NAD^+^ availability moderately increases hSOD1G93A mouse survival but also delays motor neuron degeneration, decreases markers of neuroinflammation in the spinal cord, and modifies muscle metabolism [14].

LA can also be exported out of cells through monocarboxylate transporters (MCTs) encoded by the SLC16A gene family [15]. Skeletal muscle contains MCT1 and MCT4 [16]. MCTs are the essential transporters that regulate LA flux acrossthe plasma membrane. MCT1 is ubiquitously expressed in many tissues, whereas MCT4 is present primarily in skeletal muscle [17]. Interestingly, it has been found that inhibiting MCT4 (MCT4^-/-^ mice) did not change the normal muscle morphology and contractility but induced an exercise intolerant phenotype and a significant structural degeneration of the neuromuscular junctions (NMJs). Therefore the idea that disruption of the lactate shuttle impacts motor function and destabilizes the motor unit [18] may strengthen the “lactate” hypothesis of amyotrophic lateral sclerosis proposed by VadakkadathMeethal and Atwood in 2012 [19] seems to be very interesting.

Authors have proposed that dysfunction of the adenosine triphosphate (ATP)-dependent muscle neuronal lactate shuttle (MNLS) at the NMJ that regulates the flow of LA from muscle to neurons and vice versa may be a critical factor in the pathogenesis of ALS. Failure of the MNLS due to respiratory chain dysfunction was proposed to result in LA toxicity and degeneration of nerve endings at the NMJ leading to nerve terminus dysfunction from the muscle cell. Indeed, our previous studies have shown significant decreases in oxidative metabolism in the skeletal muscles of ALS animals, especially evident during the terminal phase of the disease [5]. In the final stage of the disease, significant decreases in glycolytic enzyme activities (LDH) were also observed [5]. However, interestingly, in the spinal cord, LDH activity, after dropping at the first onset of the disease, increases its activity during the terminal stage of ALS [20]. However, it has not been investigated if changes in LDH activity are accompanied by changes in muscle and blood LA levels, or the effects of swim training on LA metabolism, the amount of NAD^+^ and NADH muscle nucleotides, as well as the activities of NADH shuttle enzymes in skeletal muscle of ALS mice. This change may underlie muscle degeneration in ALS.

The aim of this study was to determine if ALS affected lactate concentration in blood and skeletal muscle and the amount of lactate transporters (MCT1 and MCT4) during the disease progression. We also investigated if changes in LDH activity were associated with the amount of NAD^+^ and NADH muscle nucleotides and the activities of NADH shuttle enzymes in the skeletal muscle of ALS mice. Here, we hypothesize that ALS modulates LA metabolism, the amount of NAD^+^ and NADH skeletal muscle nucleotides, and the activities of MAS enzymes in the skeletal muscle of ALS mice. Furthermore, swim training at least partially reverses changes in LA metabolism in the tibialis anterior (TA) skeletal muscle.

## 2. Results

### 2.1. Effects of ALS Disease Progression and Swim Training on Skeletal Muscle LDH 2.1 and LDH 0.3 Activities and Skeletal Muscle and Blood Lactate Concentrations

**Figure 1:**
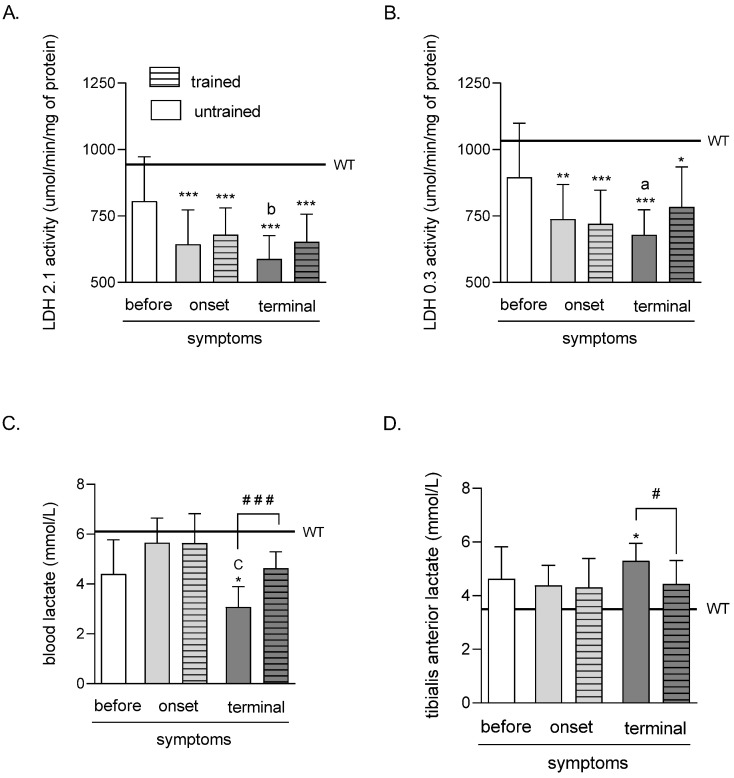
Effects of ALS disease progression and swim training on skeletal muscle LDH 2.1 and LDH 0.3 activities and skeletal muscle and blood LA concentration. LDH 2.1 (**A**) and LDH 0.3 (**B**) activities were measured in the tibialis anterior muscle of mice; LA concentration was measured in the blood (**C**) and the tibialis anterior muscle of mice (**D**). There were significant differences between the groups: * *p* < 0.05, ** *p* < 0.01 and *** *p* < 0.001 vs. WT group of mice, ^a^ *p* < 0.05 and ^b^ *p* < 0.01 vs. BEFORE group, ^C^ *p* < 0.001 vs. ONSET group (Tukey’s post-hoc test), ^#^ *p* < 0.05 and ^###^ *p* < 0.001 between indicated group (Student’s *t*-test). The data are presented as the means ± SEM (*n* = 7 in each group).

LDH 2.1 activity was significantly lower in both (trained and untrained) ONSET and TERMINAL groups of mice (643.65 ± 45.77, 679.57 ± 35.61, 588.49 ± 30.93, and 652.54 ± 36.87 μmol/min/mg of protein in ONSET untrained, ONSET trained, TERMINAL untrained, and TERMINAL trained groups of mice, respectively) than in the WT group of mice (944.08 ± 31.09 μmol/min/mg of protein). Further, LDH 2.1 activity was significantly lower in the TERMINAL untrained group than in the BEFORE group (805.76 ± 59.08 μmol/min/mg of protein) (Figure 1A).

The LDH 0.3 activity was significantly lower in both (trained and untrained) ONSET and TERMINAL groups of mice (738.27 ± 46.08, 720.01 ± 44.88, 679.14 ± 33.27, and 784.37 ± 53.09 μmol/min/mg of protein in ONSET untrained, ONSET trained, TERMINAL untrained, and TERMINAL trained groups of mice, respectively) than in the WT group of mice (1033.42 ± 34.44 μmol/min/mg of protein). Further, LDH 0.3 activity was significantly lower in the TERMINAL untrained group than in the BEFORE group (895.11 ± 72.08 μmol/min/mg of protein) (Figure 1B).

Based on the method described by [21], the LDH M and H subunit activities and total LDH and the ratio of M and H subunits were calculated. 

The total LDH activity was significantly lower in both (trained and untrained) ONSET and TERMINAL groups of mice (779.45 ± 45.73, 791.60 ± 48.12, 706.20 ± 35.60, and 799.19 ± 37.04 μmol/min/mg of protein in ONSET untrained, ONSET trained, TERMINAL untrained and TERMINAL trained groups of mice, respectively) than in the WT group of mice (1106.56 ± 35.12 μmol/min/mg of protein). Further, total LDH activity was significantly lower in the TERMINAL untrained group than in the BEFORE group (948.82 ± 72.75 μmol/min/mg of protein) (Figure S1A).

The LDH M subunit activity was significantly lower in both (trained and untrained) TERMINAL groups of mice (117.66 ± 20.97 and 166.36 ± 22.93 μmol/min/mg of protein in TERMINAL untrained and TERMINAL trained groups of mice, respectively) than in the WT group of mice (294.13 ± 36.64 μmol/min/mg of protein) (Figure S1B).

The LDH H subunit activity was significantly lower in trained ONSET and untrained TERMINAL groups of mice (560.15 ± 49.39 and 588.54 ± 30.14 μmol/min/mg of protein in ONSET trained and TERMINAL untrained groups of mice, respectively) than in the WT group of mice (812.44 ± 42.74 μmol/min/mg of protein) (Figure S1C).

There were no significant changes in the LDH H to M subunit activity ratio. However, this ratio tended to increase in the TERMINAL untrained group of mice (7.00 ± 1.66) as compared with those in the WT (3.36 ± 0.66, *p* = 0.091), BEFORE (3.31 ± 0.45, *p* = 0.083), and ONSET trained (3.21 ± 0.78, *p* = 0.071) groups of mice (Figure S1D).

The LA concentration in the blood of mice was significantly lower in the TERMINAL untrained group of mice (3.08 ± 0.29 mmol/L) than that in the WT group of mice (6.11 ± 0.57 mmol/L). Further, blood LA concentration was significantly lower in the TERMINAL untrained group than in the ONSET untrained group (5.65 ± 0.35 mmol/L). There was also a significantly higher blood LA concentration after training at the terminal stage of the disease (4.63 ± 0.23 mmol/L) than in TERMINAL untrained group of mice (Figure 1C).

The LA concentration in the TA muscle of mice was significantly higher in the TERMINAL untrained group of mice (5.30 ± 0.23 mmol/L) than that in the WT group of mice (3.49 ± 0.34 mmol/L). Further, there was a significantly lower TA muscle LA concentration after training at the terminal stage of the disease (4.43 ± 0.31 mmol/L) than that in TERMINAL untrained group of mice (Figure 1D).

### 2.2. Effects of ALS Disease Progression and Swim Training on Skeletal Muscle Amounts of Total Nicotinamide Adenine Dinucleotides (NAD^+^ and NADH), NAD^+^, NADH, and the NAD^+^/NADH Ratio

**Figure 2:**
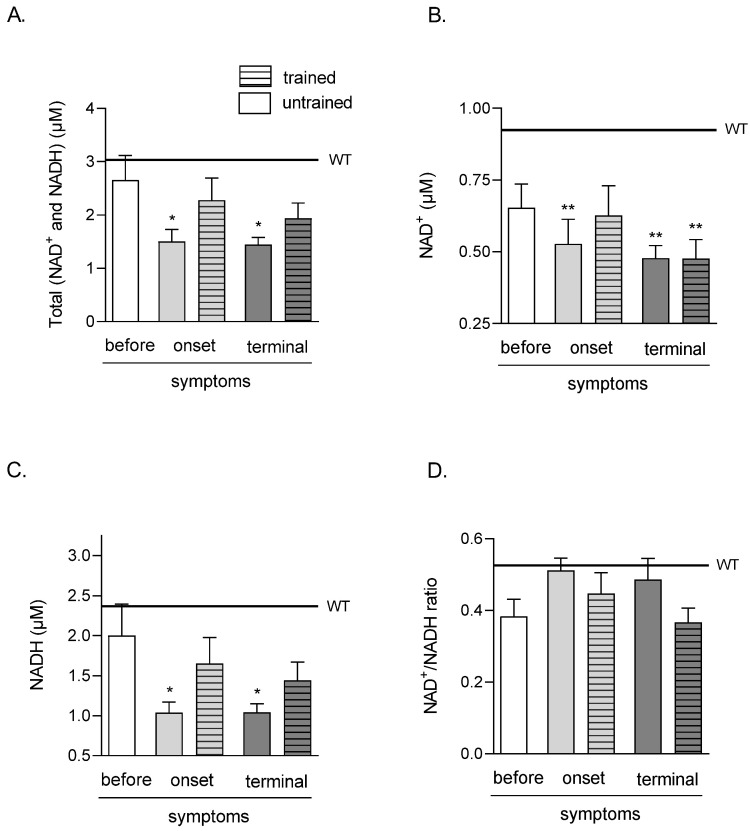
Effects of ALS disease progression and swim training on skeletal muscle amounts of total (NAD^+^ and NADH), NAD^+^, NADH, and NAD^+^/NADH ratio. Total (NAD^+^ and NADH) (**A**), NAD^+^ (**B**), NADH (**C**) NAD^+^/NADH ratio (**D**) were measured in the tibialis anterior muscle of mice. There were significant differences between the groups: * *p* < 0.05 and ** *p* < 0.01 vs. WT group of mice (Tukey’s post hoc test). The data are presented as the means ± SEM (*n* = 6 in each group).

The total amounts of TA muscle nicotinamide adenine dinucleotides (NAD^+^ and NADH) were significantly lower in both ONSET and TERMINAL untrained groups (1.51 ± 0.22 and 1.45 ± 0.13 μM, respectively) than that in the WT group of mice (3.04 ± 0.31 μM). Swim training did not significantly increase the analyzed coenzyme amounts in both groups (2.28 ± 0.42 and 1.94 ± 0.28 μM in ONSET and TERMINAL trained groups, respectively) (Figure 2A). 

The amounts of NAD^+^ in TA muscle were significantly lower in ONSET untrained and both TERMINAL (untrained and trained) groups (0.53 ± 0.08, 0.48 ± 0.04, and 0.48 ± 0.07 μM in ONSET untrained, TERMINAL untrained and trained groups of mice, respectively) than in the WT group of mice (0.92 ± 0.04 μM) (Figure 2B).

NADH in the TA muscle was significantly lower in both ONSET and TERMINAL untrained groups of mice (1.04 ± 0.13 and 1.04 ± 0.11 μM, respectively) than that in the WT group of mice (2.37 ± 0.36 μM). Swim training did not significantly increase NADH in both groups of mice (1.65 ± 0.32 and 1.44 ± 0.23 μM in ONSET and TERMINAL trained groups, respectively) (Figure 2C).

There were no NAD^+^/NADH ratio changes in all analyzed groups (Figure 2D).

### 2.3. Effects of ALS Disease Progression and Swim Training on Skeletal Muscle MDH, mMDH, cMDH Activities, and cMDH/LDH 2.1 Ratio

**Figure 3:**
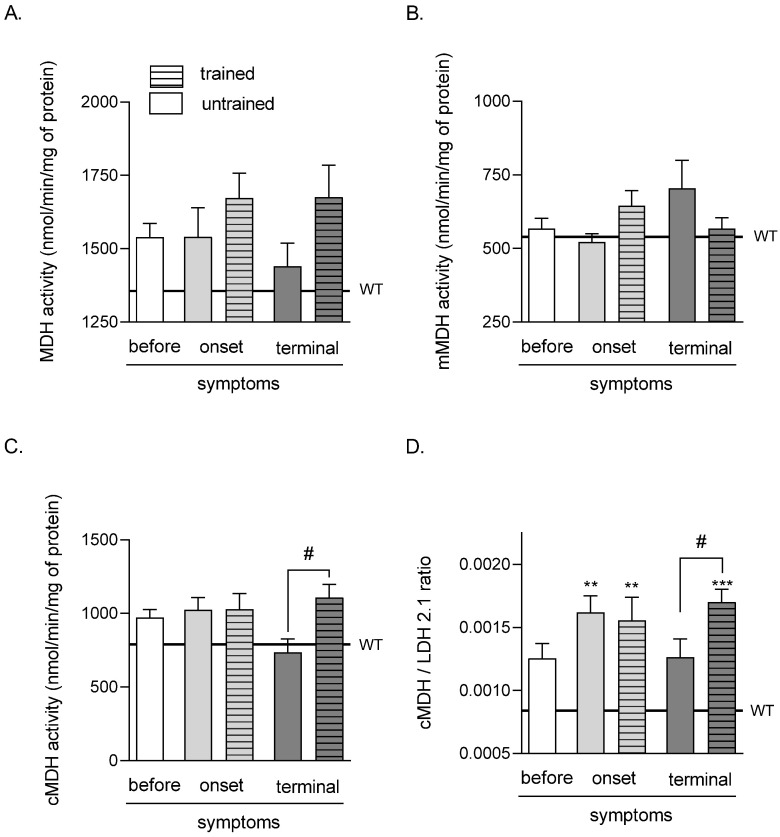
Effects of ALS disease progression and swim training on skeletal muscle MDH, mMDH, cMDH activities, and the cMDH/LDH2.1 ratio. MDH activity (**A**), mMDH activity (**B**), cMDH activity (**C**), and the cMDH/LDH2.1 ratio (**D**) were measured in the tibialis anterior muscle of mice. There were significant differences between the groups: ** *p* < 0.01 and *** *p* < 0.001 vs. WT group of mice (Tukey’s post hoc test), ^#^ *p* < 0.05 between indicated group (Student’s *t*-test). The data are presented as the means ± SEM (*n* = 7 in each group).

There were no significant differences in all analyzed groups in TA muscle MDH and mMDH activities (Figure 3A,B). Further, there were significant increases in cMDH activity after swim training in the terminal groups (735.32 ± 91.81 and 1107.68 ± 88.97 nmol/min/mg of protein in untrained and trained TERMINAL groups, respectively) (Figure 3C).

The values of the cMDH/LDH 2.1 ratio were significantly higher in both (trained and untrained) ONSET and TERMINAL trained groups of mice (0.0016 ± 0.00013, 0.0015 ± 0.00018, and 0.0017 ± 0.0001 in ONSET untrained, ONSET trained, and TERMINAL trained groups of mice, respectively) than that in the WT mice group of mice (0.001 ± 0.00004). Further, the cMDH/LDH 2.1 ratio was significantly lower in the TERMINAL untrained group (0.0013 ± 0.00014) than that in the TERMINAL trained group (Figure 3D).

### 2.4. Effects of ALS Disease Progression and Swim Training on Skeletal Muscle MCT1 and MCT4 Protein Levels

**Figure 4:**
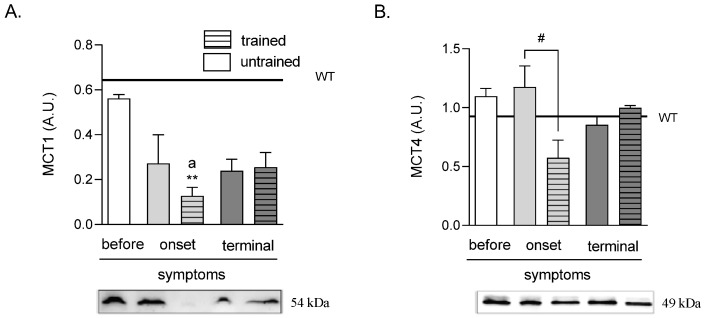
Effects of ALS disease progression and swim training on skeletal muscle MCT1 and MCT4 protein levels. MCT1 (**A**) and MCT4 (**B**) protein levels were measured in the tibialis anterior muscle of mice. There were significant differences between the groups: ** *p* < 0.01 vs. WT group of mice, ^a^ *p* < 0.05 vs. BEFORE group (Tukey’s post-hoc test), ^#^ *p* < 0.05 between indicated group (Student’s *t*-test). The data are presented as the means ± SEM (*n* = 3 in each group).

The MCT1 protein level was significantly lower in the ONSET trained group of mice (0.13 ± 0.04 A.U.) than those in the WT (0.64 ± 0.15 A.U.) and BEFORE (0.56 ± 0.02 A.U.) groups of mice (Figure 4A).

There were no changes in MCT4 protein levels in all ALS groups as compared with the WT group of mice. Further, swim training at the ONSET stage of the disease (0.58 ± 0.15 A.U.) induced a lowering of MCT4 protein level as compared with the ONSET untrained group (1.18 ± 0.18 A.U.). There was also a tendency of an increased MCT4 protein level at the terminal stage of the disease from 0.85 ± 0.07 A.U. in the TERMINAL untrained to 1.00 ± 0.01 A.U. in the TERMINAL trained groups of mice (*p* = 0.088) (Figure 4B).

### 2.5. Effects of Swim Training on the Glycolysis and NADH Shuttle Enzyme Proteomic Profiles of ALS Mice Skeletal Muscle

**Figure 5:**
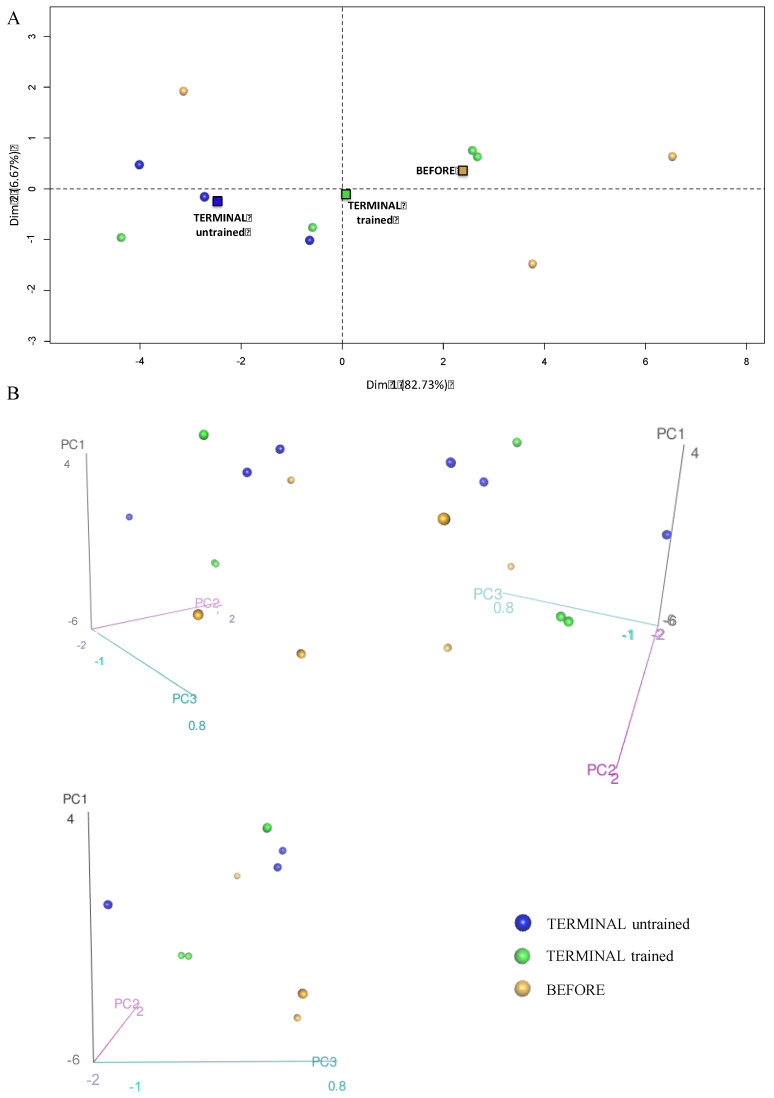
Principal component analysis showing differences in the glycolysis proteome profiles of the BEFORE, TERMINAL untrained, and TERMINAL trained groups: (**A**) The 2D graph of variables PC1 and PC2 created using a PCA based on the level of 16 identified proteins involved in glycolysis measured in TA muscle of ALS mice; (**B**) the 3D graphs of variables PC1, PC2, and PC3 created using a PCA based on the level of 16 identified proteins involved in glycolysis measured in the TA muscle of ALS mice. 3D individual graphs show the view from a different angle on the same distribution of events in the 3D space. The data are presented as the means (squares) and individual results (dots) (*n*  =  3 in each group).

**Figure 6:**
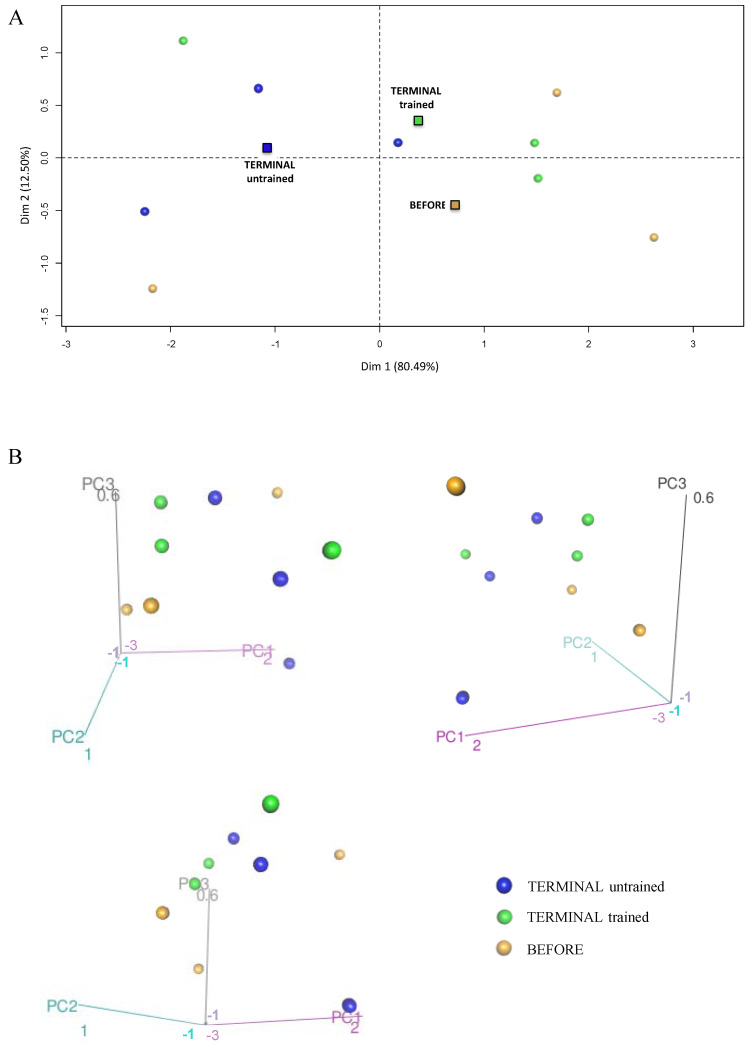
Principal component analysis presenting the profile of NADH shuttle enzymes (cASPAT, mASPAT, cMDH, and mMDH) in the skeletal muscle of the BEFORE, TERMINAL untrained, and TERMINAL trained mice: (**A**) The 2D graph of variables PC1 and PC2 created using PCA based on the level of 4 identified NADH shuttles enzymes (MAS) measured in ALS mice samples; (**B**) the 3D graphs of variables PC1, PC2, and PC3 created using PCA based on the level of 4 identified NADH shuttles enzymes (MAS) measured in ALS mice samples. 3D individual graphs show the view from a different angle on the same distribution of events in the 3D space. The data are presented as the means (squares) and individual results (dots) (*n*  =  3 in each group).

To investigate the effect of swim training on the glycolysis and NADH shuttle enzyme proteomic profiles, we took into consideration 16 proteins identified in the proteomic analysis that were related to the glycolysis process, i.e., HXK1, HXK2, G6PI, K6PF, ALDOA, TPIS, GAPDH, PGK1, PGAM2, ENOA, ENOB, B0QZL1, KPYM, KPYM1, E9Q509, and LDHA. PCA enabled the linear transformation of the 16 variables (the levels of individual above-listed proteins) into a two-dimensional space, simultaneously retaining the maximum amount of information about individual variables taken for the analysis. The newly created PCA variables, PC1 and PC2, indicated possible differences in the “glycolysis proteomic profiles” among the BEFORE, TERMINAL untrained, and TERMINAL trained groups (Figure 5A). Much better results were obtained using the three newly created PCA variables (PC1, PC2, and PC3), i.e., 3D graphs from above listed 16 identified proteins. We found that the BEFORE glycolytic signature (fingerprint created based on the proteomic profile) seemed to be different from those obtained for the TERMINAL untrained and TERMINAL trained groups. Interestingly, a similar analysis performed for the four identified in the proteome NADH shuttle enzymes (cASPAT, mASPAT, cMDH, and mMDH) revealed no differences between the obtained profiles (Figure 6A,B). The lack of differences in the profiles of cASPAT, mASPAT, cMDH, and mMDH means that swim training has no effect on the level of NADH shuttle enzymes when they are analyzed globally. A separate analysis of individual proteins also revealed any changes in the level of investigated proteins. Moreover, the unchanged levels of mMDH and cMDH support the lack of differences in the measured (total) MDH activity (Figure 3A). The increased activity of cMDH in the TERMINAL trained mice as compared with the untrained counterparts can not be explained by the changes in the level of cMDH level, which is stable, based on the proteomic analysis.

## 3. Discussion

### 3.1. Effect of ALS Disease on Lactate Metabolism

An important, although unexpected and challenging to explain, phenomenon observed in our study is the discrepancy between the accumulation of LA in TA muscle and the activity of the LDH enzyme as an indicator of the glycolytic pathway in a group of ALS mice during the terminal stage of the disease. Generally, first, increased LA concentration in the skeletal muscles results in greater LA production, thus, accelerating the glycolytic pathway. Second, it may be caused by a disruption in the LA transport into and out of the muscle cells. Third, it may result from dysfunction in LA oxidation in skeletal muscles. Fourth, it appears that it may be related to the phenomenon of skeletal muscle damage, as discussed below.

However, the accumulation of LA in TA muscle in ALS mice is intriguing because of the lower activity of the LDH 2.1 enzyme, which is responsible for the production of lactic acid in muscle cells. Our previous studies that used the thigh muscle also documented a decrease in LDH 2.1 and LDH 0.3 activities, but we did not link this to lactate metabolism or investigate the reason for a decrease in these activities. Meanwhile, another team also documented the reduced activities of glyceraldehyde-3-phosphate dehydrogenase (GAPDH) in the skeletal muscles of ALS mice [3]. The changes observed in LDH activity and glycolytic protein proteome analysis suggested that the dysfunction in the glycolytic process was a crucial variable during ALS progression in skeletal muscle. In order to investigate the reasons for glycolysis inhibition, we decided to measure the concentration of NAD^+^ and NADH nucleotides. As shown, the activity of GAPDH, the key glycolytic enzyme, depends on the availability of the NAD^+^ pool. NAD^+^ limitation inhibits the activity of GAPDH and the course of glycolysis [22]. Interestingly, the concentrations of the NAD^+^ and NADH nucleotide pools turned out to be significantly lower in ALS mice from the first symptoms of ALS than in mice during the terminal stage of the disease. Similarly, the concentration of NAD^+^ alone was most reduced there, which may explain the reduced activities of glycolytic enzymes, including LDH, in the skeletal muscle of ALS mice. 

There are two mechanisms to rebuild the concentration of NAD^+^ in muscle cells. The first is the oxidation of NADH to NAD^+^ in a reaction catalyzed by M-LDH. Because we observed reduced activity of this enzyme in skeletal muscle in ALS mice, we decided to investigate an alternative route of NAD^+^ recovery, the activity of NADH transporting enzymes, including malate dehydrogenase (total, mMDH, and cMDH). cMDH competes with LDH 2.1 for NADH and, at the same time, indirectly interacts with this enzyme because increasing the cytosolic NAD^+^ pool in muscle cells creates a chance to accelerate the glycolytic pathway. However, the lowest cMDH activities in TA muscle seen in ALS mice during the terminal stage of the disease indicate a breakdown of this alternative pathway. Therefore, these results suggest that the lack of a mechanism to restore the NAD^+^ pool may inhibit glycolysis in the TA muscle of ALS mice. Inhibition of glycolysis in mice with ALS contradicts the explanation for the increased LA concentration in TA muscle.

The second explanation for the observed changes relates to the potential disruption in LA transport through the sarcolemma. As mentioned earlier, the MCT1 and MCT4 transporters in skeletal muscle are responsible for LA uptake from the circulation and LA extrusion out of muscle cells, where lactate uptake from the circulation is highly correlated with the MCT1 content in muscles [17]. However, reduced amounts of MCT1 protein are seen in both of the groups of ALS mice (ONSET and TERMINAL). It has been documented that MCT1 expression correlates highly with oxidative metabolism [17,23]. MCT1 expression also increases due to training or chronic electrical stimulation of rat hindlimb muscles [17,24,25] and decreases, for example, due to the denervation of rat hind limb muscles [26], which indirectly relates to the course of ALS disease. Therefore, our results may indicate that decreases in the contractile activity of skeletal muscles, progressing with the development of the disease, may contribute to a decrease in the expression of MCT1 protein. 

The deletion of MCT4 does not change the normal muscle morphology and contractility but does induce a significant structural degeneration of NMJ [18]. Therefore, we decided to investigate MCT4 protein expression in the TA muscle of ALS mice. Contrary to expectations, we did not observe differences in the expression of this protein between the groups of ALS mice nor between the groups of ALS and control mice. Moreover, MCT4 is responsible for LA efflux from muscle cells [17]. Thus, the lack of visible changes in MCT4 protein concentrations in the TA muscles of ALS mice may indicate a different pathomechanism responsible for LA accumulation in these animals. However, the lack of changes in the amount of MCT4 does not necessarily explain the activity of this transporter, which, despite the unchanged amount, may not function properly, which requires further study. Inhibition of MCT1 and MCT4 increases the amount of LA in the muscle [27]. This dysfunction of the LA transport system probably explains the higher LA concentration in the skeletal muscle of ALS mice.

Therefore, we set out to investigate an alternative pathway of muscle LA degradation and measure the activity of the LDH 0.3 enzyme, characteristic of the H4 enzyme subunit, which converts lactate to pyruvate, enabling its further oxidation in the mitochondria to CO_2_ and H_2_O. Interestingly, the lowest activities of this enzyme were in the TA muscle of the terminal group of ALS mice, which also had the highest accumulation of LA. Thus, the decreased activity of the LDH 0.3 enzyme seems to be a significant factor responsible for the disruption of LA metabolism by limiting the possibility of LA oxidation in muscle cells.

Fourth, the last possibility is leakage of the LDH enzyme due to damage to the muscle cell membranes in terminal ALS mice. Our blood creatine kinase (CK) activity results confirm this possibility, showing the greatest muscle damage in ALS mice at this stage [8].

### 3.2. Effect of Swim Training on Lactate Metabolism in ALS Mice

Our research and other teams have shown that swim training could bring many benefits in a mouse model of ALS disease, including extending the lifespan of ALS mice [5,28]. We have demonstrated, among others, that it reduced body weight loss, skeletal muscle mass, and oxidative stress, and it improved muscle bioenergetics [4,5,8]. Moreover, Desseille et al. showed that swim training reduced drops in blood LA concentrations (2017), similar to the results of our research. However, so far, the effect of training on skeletal muscle LA metabolism has not been studied. Contrary to expectations, swim training did not significantly affect the MCT 1 and MCT 4 transport protein levels. This proved that the explanation of changes in LA concentrations in skeletal muscles should be associated with the possibility of LA oxidation. Swim training partially reversed the changes in LA concentrations in ALS mice by increasing blood LA concentrations and decreasing skeletal muscle LA concentrations in terminal ALS mice. Interestingly, although not statistically significant, these changes were accompanied by increases in the activities of both LDH 2.1 and LDH 0.3 enzymes, responsible for the production and oxidation of LA in TA muscle. Although not statistically significant, an upward trend was also observed in the NAD^+^ and NADH nucleotide pools and NADH itself after training in both early-symptomatic and terminal ALS mice. Increasing the pool of nucleotides has been shown to be beneficial for cellular metabolism and the course of ALS disease as it may accelerate glycolysis and slow down the disease’s negative symptoms, as mentioned above [14,22]. Significant effects of swim training were visible in the case of the cMDH enzyme, which, alternatively to LDH 2.1, is responsible for the oxidation of NADH generated during glycolysis and the restoration of NAD^+^. It seems that this enzyme, along with mMDH, especially during the terminal stage of ALS disease, becomes alternative protection of the nucleotide pool and a source of additional ATP molecules generated on the respiratory chain in mitochondria in skeletal muscles. The cMDH/LDH 2.1 ratio confirmed the increasing role of cMDH as the disease progresses. The results of total MDH and its isoform activities differed from those obtained in the thigh muscle of mice with the first symptoms of ALS [4]. These differences may result from differences in the muscle fiber type composition between these muscles [29,30,31]. Another explanation for these differences may be the greater hyperlocomotion of these mice, which is seen early in life until the first symptoms of the disease appear, i.e., around 16 weeks of age [20]. The observed changes in the glycolysis process are in line with proteomic analysis; however, the unchanged levels of mMDH and cMDH support the lack of differences in the measured (total) MDH activity (Figure 3A). The increased activity of cMDH in TERMINAL trained mice as compared with the untrained counterparts can not be explained by the changes in the level of cMDH, which was stable, based on the proteomic analysis.

Although SOD1 G93A mice are still a gold standard in preclinical studies of ALS disease, additional studies are needed to confirm the obtained results on other ALS models that also include other gene mutations associated with ALS. Searching for new mechanisms induced by swim training to improve ALS lifespan and quality of life might help treat ALS and other neuromuscular diseases.

## 4. Materials and Methods

### 4.1. Animals

Transgenic male mice with the G93A human SOD1 mutation B6SJL-Tg (SOD1G93A) 1Gur/J (ALS mice) (*n* = 35), and wild-type male mice B6SJL (*n* = 7) were purchased from The Jackson Laboratory (Bar Harbor, ME, USA). The mice were housed in an environmentally controlled room (23 ± 1 °C with a 12 h light/dark cycle); the mice received standard chow and water ad libitum. After acclimatization, the mice were randomly divided into the following groups according to disease progression and training status as previously described [4,5]: ALS BEFORE, ALS untrained mice with no visible signs of the disease; ALS ONSET, ALS untrained and trained with first symptoms disease; ALS TERMINAL, untrained and trained in the last stage of disease; and the wild-type (WT) group of mice. The mice were euthanized by cervical dislocation. The mice from the ALS BEFORE and WT groups of mice were euthanized on the 10th week of life. The mice from the ALS ONSET group were euthanized when we observed the first symptoms of disease (16th week of life), and the mice from the ALS TERMINAL group were euthanized when we observed the last stage of disease (19th week of life), as described by [8].

### 4.2. Swim Training Protocol 

Starting at 10 weeks of age, the transgenic groups of ALS mice (ONSET TRAINED and ALS TERMINAL TRAINED) underwent the training procedure according to Deforges et al. [28] with the slight modification described by Flis et al. [5]. The swim training was conducted in a unique pool with regulated water flow, 5 times a week for 30 min. The water temperature was 30 °C, and the maximum flow speed was 5 L/min. At 105 days of life, the training frequency was reduced to 3 times a week. The exercise time and water flow were adapted to the individual possibility of the ALS mice. The training was continued to 115 days of age.

### 4.3. Tissue Homogenization and Lysate Preparation

Following the dissection performed at 4 °C, the tibialis anterior muscle samples were rapidly removed, trimmed of visible connective tissue, weighed, frozen in liquid nitrogen, and kept at −80 °C until further analysis. 

#### 4.3.1. Preparation for the Western Blotting Analysis in the Tibialis Anterior Muscle

Next, the samples were homogenized using a Bio-Plex Pro ™ Cell Signaling Reagent Kit (Bio-Rad, Hercules, CA, USA, catalog # 171-304006M) with minor modifications. Tissue (about 20 mg) was placed in 1.5 mL Eppendorf and treated with 100 µL cell wash buffer. After rinsing and draining, the material was cut and transferred to 2 mL Eppendorf, and 200 µL cell lysis buffer was added to the material and was homogenized manually 20 times. The lysis buffer was prepared according to the instructions and contained a protease cocktail (Sigma P8340). The lysis buffer composition per 1.02 mL was: 1 mL of cell lysis buffer, 10 µL cell lysis factor QG, and 10 µL cocktail of protease and phosphatase inhibitors. Then, each lysate was frozen (−70 °C) and thawed (30 °C) three times, and re-homogenized (10 times). Finally, the material was centrifuged at 15,000× *g* for 10 min at 4 °C (Sigma 3K30 centrifuge). The resulting supernatant was decanted and frozen for further measurements. 

#### 4.3.2. Preparation for the Nicotinamide Adenine Dinucleotides (NAD^+^ and NADH) and Lactate Analysis in the Tibialis Anterior Muscle

All procedures were conducted according to an NAD^+^/NADH Assay Kit (Abcam, ab176723) using chemicals supplied by the manufacturer. Briefly, tissues (20 mg of tibialis anterior muscle) were washed with cold PBS and homogenized in a 400 µL lysis buffer. Then, homogenates were centrifuged at 2500 rpm for 10 min at room temperature. The supernatants were used to measure nicotinamide adenine dinucleotides (NAD^+^ and NADH) and LA. 

#### 4.3.3. Preparation for the Analysis of Enzyme Activities in the Tibialis Anterior Muscle

After thawing, skeletal muscle was weighed and immediately inserted into cold homogenization buffer (50 mM potassium phosphate, 1 mM EDTA, 0.5 mM DTT, 1.15% KCl, pH 7.4, supplemented with protease inhibitor cocktail). Next, 5% homogenate was made in a hand-glass homogenizer. After obtaining the homogenate (around 30 stokes), the homogenate was immediately frozen and kept at −80 °C until analysis.

### 4.4. Blood Collection and Preparation

Blood was collected for lactate measurement from the heart of mice into tubes containing EDTA, and then centrifuged 2000× *g* for 10 min. The plasma thus obtained was used for all analyses.

### 4.5. Measurement of Enzymes Activities 

All enzyme activities were measured spectrophotometrically (Cecil CE9200, Cecil Instruments Limited, Cambridge, UK) in tibialis anterior muscles homogenates. 

#### 4.5.1. Malate Dehydrogenase

The malate dehydrogenase (MDH) activity was measured at 30 °C, according to [11]. Briefly, 10 µL of homogenate (1:10, 5%) was incubated for 2 min in 970 µL of buffer (50 mM Tris-HCl, 5 mM EDTA, pH 7.6) supplemented with 10 µL of freshly made NADH (20 mM). Next, 10 µL of freshly made oxaloacetic acid (20 mM) was added to initiate the reaction. The reactions were conducted in duplicate, and absorbance was read at 340 nm. The mitochondrial MDH (mMDH) activity was assessed after 2 min preincubation of 20 µL homogenate (1:10, 5%) with 98% ethanol (1:1 vol). The cytosolic MDH (cMDH) was calculated as the difference between MDH and mMDH activities. The enzyme activities are expressed as nmol/minute/mg of protein. 

#### 4.5.2. Lactate Dehydrogenase

The lactate dehydrogenase (LDH) activity was measured at 30 °C, according to [21]. Two concentrations of pyruvate (PYR) were used to determine the maximal activity of lactate dehydrogenase characteristics for subunit M4 (LDH 2.1) in the presence of 2.1 mM PYR and subunit H4 (LDH 0.3) in the presence of 0.3 mM PYR. After 2 min preincubation of tissue (10 µL homogenate (1:10, 5%)) with buffer (970 µL, 50 mM KPi, 1 mM EDTA, pH 7.4), the substrates NADH and pyruvate were added immediately before the measurement of the enzyme activity, and the reaction was started. The final volume in the cuvette was 1000 μL. The decrease in absorbance at 340 nm was followed for 2 min. The enzyme activities are expressed as µmol/minute/mg of protein. 

The total LDH, LDH M, and LDH H subunits were calculated, as previously described [21].

### 4.6. Lactate Measurement 

Lactate concentration was measured fluorometrically (GloMax-Multi+, Promega Corporation; Madison, WI, USA) at 25 °C, according to [32]. Briefly, 5 μL of plasma and 10 μL skeletal muscle homogenate, or standard (lithium lactate in concentration between 0–10 mmol/L), was incubated 30 min with 250 μL of the mixture containing: buffer (1.1 M hydrazine, pH 9.0, 1.3 g hydrazine sulfate, 5.0 g hydrazine hydrate, 0.2 g Na_2_EDTA, distilled water to 100 mL), 1 U/mL LDH, and 5 mmol/L NAD^+^. After incubation, the fluorescence was measured at Ex = 365 nm and Em = 410–460 nm. The lactate concentration is expressed as mmol/L in the blood and mmol/kg wet mass in skeletal muscle.

### 4.7. Total Amount of Nicotinamide Adenine Dinucleotide (NADH and NAD^+^) Measurements

The total amount of nicotinamide adenine dinucleotide (NAD^+^ and NADH) levels were conducted according to an NAD^+^/NADH Assay Kit (Abcam, ab176723, Cambridge, UK). Briefly, 25 μL of the sample, blank control, or standard were added to 25 μL of specific control or NAD^+^ extraction solutions. After 15 min of incubation at 37 °C, another 25 μL of control solution or NADH extraction solution (to neutralize NAD^+^ extracts) was added. Next, 75 μL of NADH reaction mixture was added to each well. After incubation (30 min at room temperature in the dark), fluorescence was measured at Ex = 525 nm and Em = 580–640 nm in a GloMax-Multi+ System (Promega Corporation; Madison, WI, USA). The amount of NADH was calculated by the formula: total (NAD^+^ and NADH) minus NAD^+^. The concentration of NAD^+^, NADH, and total are expressed as μM.

### 4.8. Western Blotting Analysis of MCT1 and MCT4

Equal amounts of muscle lysates (50 µg of protein per sample) were separated on 4–20% SDS-polyacrylamide gradient gels and transferred onto a polyvinylidene difluoride membrane. The analysis procedure was performed as previously described by [7]. After transfer, membranes were blocked in an EveryBlot Blocking Buffer (cat. no. 12010020, Bio-Rad, Hercules, CA, USA) for 15 min at room temperature. Next, membranes were incubated overnight at 4 °C with primary antibodies. The following antibodies were used: rabbit polyclonal IgG anti-MCT1/monocarboxylic acid transporter 1 (cat. no. ab93048, 1:1000, Abcam, Cambridge, UK); rabbit IgG anti-SLC16A3/MCT 4 (cat. no. Ab74109-100 1:1000, Abcam, Cambridge, UK). After washing (3 × 10 min) in 1 × TBST, the membranes were incubated for 1 h at room temperature with gentle shaking with anti-rabbit IgG–peroxidase conjugate (cat. no. A9169, 1:25,000, Sigma Aldrich, St. Louis, MI, USA). After incubation, immunoblots were detected and visualized using enhanced chemiluminescence reagents (Pierce, Termo Fisher Scientifc, Inc., Waltham, MA, USA). Changes in protein levels were assessed by densitometry of the immunoreactive bands and normalized to the total amount of protein in the samples transferred onto the membrane. Relative protein levels were analyzed and quantified using a ChemiDoc image analysis system (Bio-Rad Laboratories, Inc.). The immunoblotting analyses were performed for three randomly selected animals from each group.

### 4.9. Proteomic Analysis

The levels of investigated proteins (16 identified proteins related to the glycolysis process: HXK1, HXK2, G6PI, K6PF, ALDOA, TPIS, GAPDH, PGK1, PGAM2, ENOA, ENOB, B0QZL1, KPYM, KPYM1, E9Q509, and LDHA) and 4 NADH shuttle enzymes (cASPAT, mASPAT, cMDH, and mMDH) were determined in ALS BEFORE, ALS TERMINAL, untrained and trained groups, using liquid chromatography-MS3 spectrometry (LC-MS/MS) at the Thermo Fisher Center for Multiplexed Proteomics (Department of Cell Biology, Harvard Medical School, Cambridge, MA, USA). The samples were prepared as previously described by [33]. Peptide fractions were analyzed using an LC-MS3 data collection strategy and an Orbitrap Fusion mass spectrometer (Thermo Fisher Scientific Inc., Waltham, MA, USA). 

### 4.10. Data Analysis

Statistical analyses were performed using the software package Statistica v. 13.0 (StatSoft Inc., Tulsa, OK, USA). The results are expressed as the mean ± standard error of the mean (SEM). The differences associated with disease progression and between the ALS and WT groups of mice were analyzed using a one-way analysis of variance (ANOVA). If a difference was detected in these test models, the significance level was determined using Tukey’s post hoc test. To verify the significance of small, swim training-associated changes (ONSET untrained vs. trained and TERMINAL untrained vs. trained), Student’s *t*-test was used. The results were considered statistically significant at *p* < 0.05. For proteomic data, a principal component analysis (PCA) was performed using the R Statistical Software (Foundation for Statistical Computing, Vienna, Austria), a free software environment for statistical computing and graphics to visualize the similarities and differences between the studied groups.

## 5. Conclusions

In conclusion, the presented data indicate that ALS, during the terminal stage of the disease, induced a disruption in LA concentration and NAD^+^ and NADH dinucleotide pools. ALS also caused a significant reduction in the levels of the MCT1 transporter protein and decreased LDH activity in the TA muscle. Swim training partially reversed these changes, which was related to the influence of training on LDH activity and MDH enzymes and not on the level of MCT1 and MCT4 proteins.

The findings of this study shed light on the new metabolic disturbances and swimming-induced compensation of energy demands through the pathophysiological adaptation of LA metabolism in skeletal muscle. 

## Data Availability

The data presented in this study are available on request from the corresponding authors.

## Supplementary Materials

The supporting information can be downloaded at: https://www.mdpi.com/article/10.3390/ijms231911504/s1.
