# Supplementary material for: Swim Training Affects on Muscle Lactate Metabolism, Nicotinamide Adenine Dinucleotides Concentration, and the Activity of NADH Shuttle Enzymes in a Mouse Model of Amyotrophic Lateral Sclerosis
Source: Int J Mol Sci. 2022 Sep 29;23(19):11504. doi: 10.3390/ijms231911504 (PMC9569676; doi:10.3390/ijms231911504)
Supplement: Supplementary file 1 [file ijms-23-11504-s001.zip › ijms-1888180-supplementary.pdf]

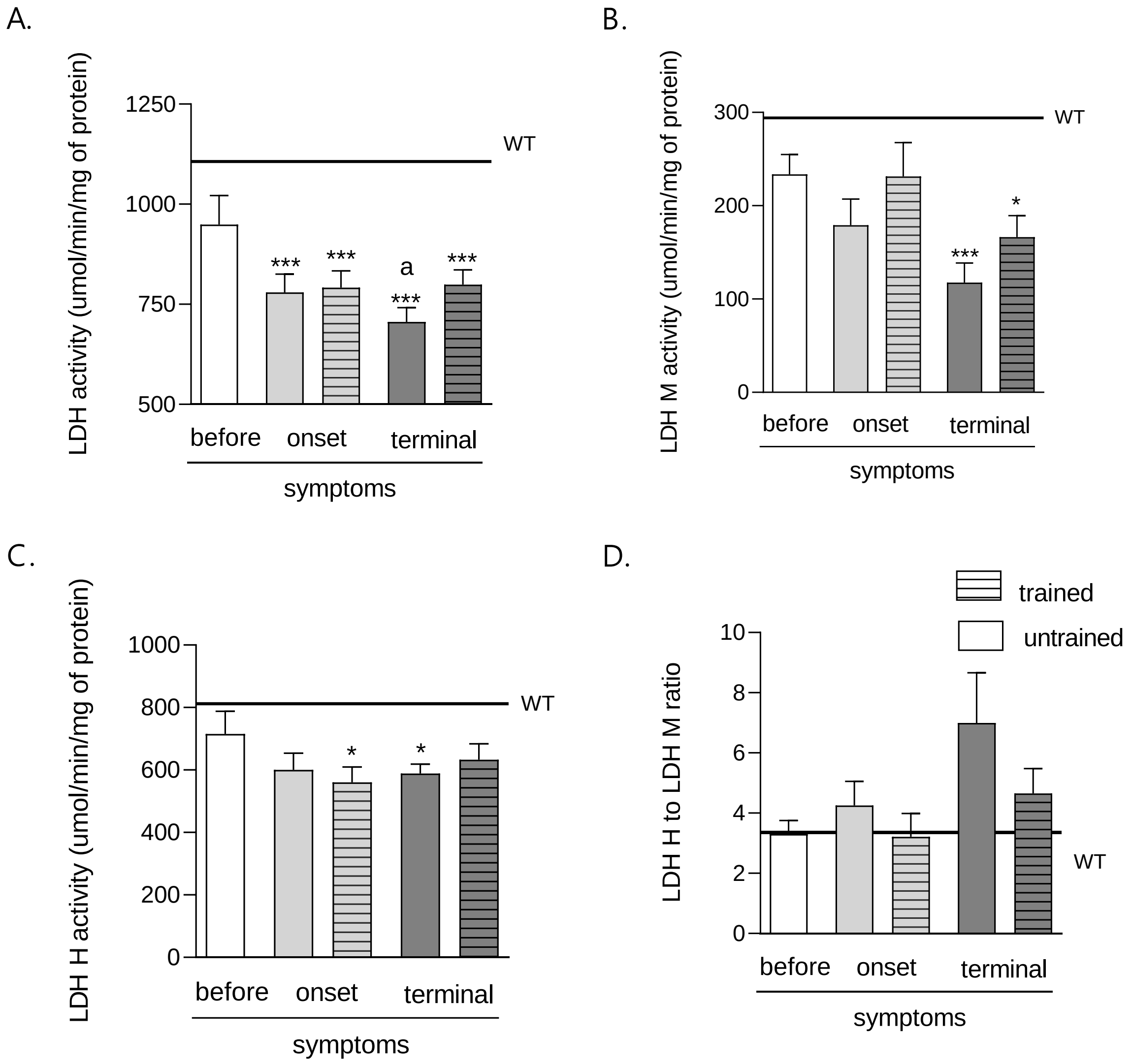

Figure S1. Effects of ALS disease progression and swim training on skeletal muscle total LDH, LDH H and M subunits activities and LDH H to M subunits ratio.

Total LDH (A), LDH H to M subunits ratio (B), LDH M subunit (C), and LDH H subunit (D) were measured in the mice *tibialis anterior* muscle. There were significant differences between the groups: *$p<0.05$, ***$p<0.001$ vs. WT group of mice, $^{a}p<0.05$, vs. BEFORE group (Tukey's post-hoc test). The data are presented as the means ± SEM (n = 7 in each group).
